# High-efficiency thermoelectric Ba_8_Cu_14_Ge_6_P_26_: bridging the gap between tetrel-based and tetrel-free clathrates†
†Electronic supplementary information (ESI) available: Calculated and experimental powder XRD patterns; tables with crystallographic information; SEM and EDS results; additional DSC, thermal conductivities, and *ZT* figures. CCDC 1568106. For ESI and crystallographic data in CIF or other electronic format see DOI: 10.1039/c7sc03482b


**DOI:** 10.1039/c7sc03482b

**Published:** 2017-09-29

**Authors:** Jian Wang, Oleg I. Lebedev, Kathleen Lee, Juli-Anna Dolyniuk, Peter Klavins, Sabah Bux, Kirill Kovnir

**Affiliations:** a Department of Chemistry , Iowa State University , Ames , Iowa 50011 , USA; b Department of Chemistry , University of California , Davis , CA 95616 , USA . Email: kovnir@iastate.edu; c Laboratoire CRISMAT , ENSICAEN , CNRS , UMR 6508 , F-14050 Caen , France; d Thermal Energy Conversion Research and Advancement Group , Jet Propulsion Laboratory , Pasadena , CA 91109 , USA; e Department of Physics , University of California , Davis , CA 95616 , USA; f Ames Laboratory , Iowa State University , Ames , Iowa 50011 , USA

## Abstract

A new type-I clathrate, Ba_8_Cu_14_Ge_6_P_26_, was synthesized by solid-state methods as a polycrystalline powder and grown as a cm-sized single crystal *via* the vertical Bridgman method. Single-crystal and powder X-ray diffraction show that Ba_8_Cu_14_Ge_6_P_26_ crystallizes in the cubic space group *Pm*3*n* (no. 223). Ba_8_Cu_14_Ge_6_P_26_ is the first representative of anionic clathrates whose framework is composed of three atom types of very different chemical natures: a transition metal, tetrel element, and pnicogen. Uniform distribution of the Cu, Ge, and P atoms over the framework sites and the absence of any superstructural or local ordering in Ba_8_Cu_14_Ge_6_P_26_ were confirmed by synchrotron X-ray diffraction, electron diffraction and high-angle annular dark field scanning transmission electron microscopy, and neutron and X-ray pair distribution function analyses. Characterization of the transport properties demonstrate that Ba_8_Cu_14_Ge_6_P_26_ is a p-type semiconductor with an intrinsically low thermal conductivity of 0.72 W m^–1^ K^–1^ at 812 K. The thermoelectric figure of merit, *ZT*, for a slice of the Bridgman-grown crystal of Ba_8_Cu_14_Ge_6_P_26_ approaches 0.63 at 812 K due to a high power factor of 5.62 μW cm^–1^ K^–2^. The thermoelectric efficiency of Ba_8_Cu_14_Ge_6_P_26_ is on par with the best optimized p-type Ge-based clathrates and outperforms the majority of clathrates in the 700–850 K temperature region, including all tetrel-free clathrates. Ba_8_Cu_14_Ge_6_P_26_ expands clathrate chemistry by bridging conventional tetrel-based and tetrel-free clathrates. Advanced transport properties, in combination with earth-abundant framework elements and congruent melting make Ba_8_Cu_14_Ge_6_P_26_ a strong candidate as a novel and efficient thermoelectric material.

## Abstract 2

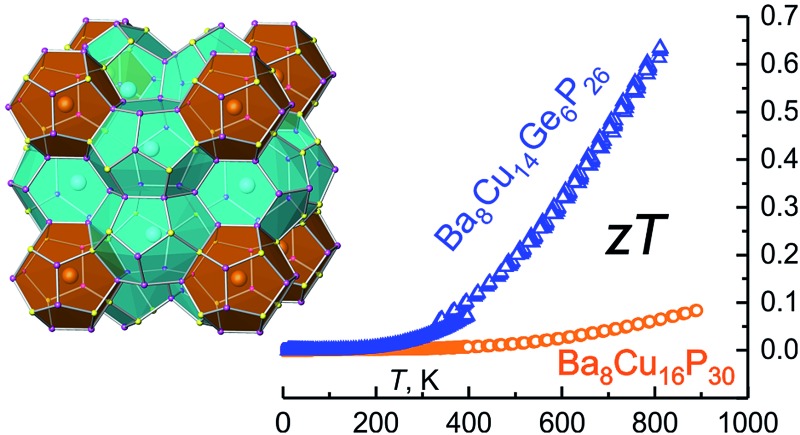
Synergy between tetrel- and pnictide-based clathrates: synthesis, crystal structure, and transport properties of a Ba_8_Cu_14_Ge_6_P_26_.

## Introduction

Thermoelectric materials play an important role in renewable energy applications, which can convert waste heat into electrical energy and *vice versa*.^1^–^3^ The efficiency of thermoelectric materials is characterized by the dimensionless figure of merit, *ZT* = *S*^2^*T*/*ρκ*, where *S* is the Seebeck thermopower, *T* is the absolute temperature, *ρ* is the electrical resistivity, and *κ* is the thermal conductivity. In crystalline solids, the intrinsic correlations between these three transport properties make enhancement of thermoelectric efficiency challenging due to their dependence on carrier concentration and electronic structure.^4^–^9^ The Phonon Glass-Electron Crystal (PGEC) concept was proposed as a way to guide thermoelectric research.^10^–^13^ Clathrate compounds, also known as host–guest compounds, are considered to be PGEC compounds due to their covalent three-dimensional frameworks and rattling of guest atoms. The framework acts as a good electrical conductor, while the rattling of guest atoms encapsulated in the framework cages efficiently scatters phonons to reduce thermal conductivity.^12^,^14^–^23^ Besides thermoelectric applications, clathrates are used as photovoltaic materials, Li-batteries anodes, superconductors, and gas storage materials.^16^,^17^

Based on the framework-forming elements, all anionic clathrates can be divided into two main groups: tetrel-based (Si, Ge, Sn) and tetrel-free clathrates. The latter group, which is much smaller than the former one, can also be called pnicogen-based or transition metal-based clathrates since the frameworks of all reported tetrel-free clathrates are formed by a combination of the late transition metals of group 10–12 with pnicogen atoms: P, As, or Sb.^17^ Both groups have distinct chemical and structural properties. One specific feature of tetrel clathrates is the formation of locally disordered frameworks where one crystallographic position is jointly occupied by different atoms, such as Ga/Ge or Zn/Sn.^16^,^17^ In turn, tetrel-free clathrates show full segregation of transition metal and phosphorus atoms over different framework positions, which result in the formation of either long-range ordered structures, as in the case of Ba_8_M_16_P_30_, BaM_2_P_4_, and Ba_8_Zn_11_Cu_13_P_28+*δ*_ (M = Cu, Au, Ni), or short-range ordering, as in the case of Ba_8_(Cu/Zn)_16+*y*_P_30–*y*_.^15^,^18^,^24^–^26^

In this work, we report a new type-I clathrate, Ba_8_Cu_14_Ge_6_P_26_, which combines a transition metal, tetrel element, and pnicogen in its framework. This compound was synthesized and grown as a large crystal through the Bridgman technique. A uniform distribution of the constituent elements and the absence of short- or long-range ordering in the Ba_8_Cu_14_Ge_6_P_26_ clathrate was confirmed by a combination of synchrotron powder X-ray diffraction, high-angle annular dark field scanning transmission electron microscopy, and X-ray and neutron pair distribution function analyses. Thermoelectric measurements demonstrate that Ba_8_Cu_14_Ge_6_P_26_ is a p-type semiconductor with a remarkable figure of merit. This newly discovered clathrate builds a bridge between the two clathrate families.

## Experimental section

### Synthesis

All preparation and handling of samples were performed in an argon-filled glovebox with the O_2_ level below 1 ppm. All starting materials were commercial grade and were used as received: Ba chunks (Sigma-Aldrich, 99.9%), Cu powder (Sigma-Aldrich, 99.9%), Ge pieces (Alfa Aesar, 99.9999%) and red P powder (Alfa Aesar, 99%).

**Fig. 1:**
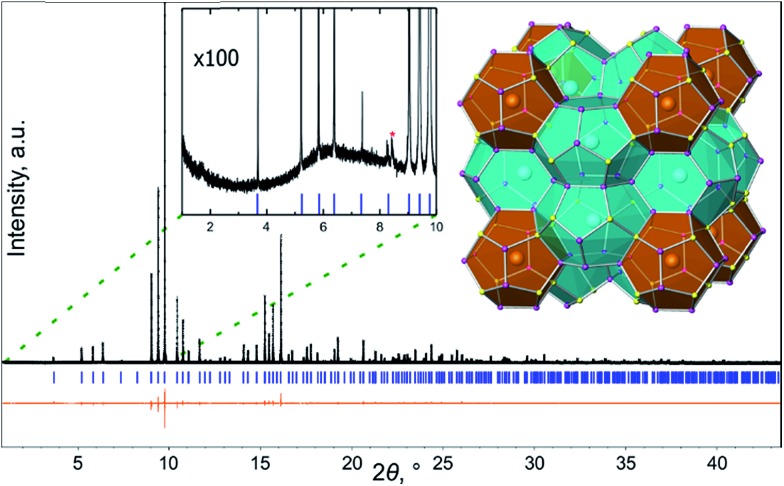
Results of Rietveld refinement of the synchrotron powder X-ray diffraction data of Ba_8_Cu_14_Ge_6_P_26_. Experimental pattern: black crosses; calculated pattern: black line; calculated peak positions: blue sticks; difference curve: orange line. Left inset: enhanced view of the low angle part of the experimental pattern with the calculated peak positions shown as blue sticks. One admixture peak is indicated with red star. Right inset: general view of the crystal structure with two types of polyhedra highlighted in (pentagonal dodecahedra) brown and (tetrakaidecahedra) cyan.

The polycrystalline sample of Ba_8_Cu_14_Ge_6_P_26_ was obtained *via* the solid-state reaction of the elements. The elements with a stoichiometric Ba/Cu/Ge/P ratio of 8/14/6/26 were loaded into a carbonized silica ampoule, evacuated, and flame-sealed. The ampoule was first heated from room temperature to 1123 K over 17 h, and then it was annealed at this temperature for 144 hours. After the furnace was turned off and cooled, the samples were ground and reloaded into a new carbonized silica ampoule in the glovebox, resealed, and reannealed using the same temperature profile as the first annealing. The same procedure was repeated a third time. After three annealings, a uniform melted chunk was found as the final product, and was characterized as single-phase by powder X-ray diffraction (Fig. 1 and S1†).

### Differential scanning calorimetry

A Netzsch Differential Scanning Calorimeter (DSC) was used to characterize the thermal behavior of Ba_8_Cu_14_Ge_6_P_26_. A powder sample (mass: 35.3 mg) was sealed inside an evacuated silica ampoule, heated to 1273 K and cooled down to room temperature with the 10 K min^–1^ rate.

### Bridgman growth

About 5 g of single-phase polycrystalline sample was ground into fine powder in a mortar and loaded into a carbonized silica ampoules with a sharp cone end. The ampoule was evacuated and flame-sealed. This ampoule was further sealed in another thick-walled silica ampoule, which was used to protect the growth tube. The silica ampoule was placed into a home-made two-zone vertical Bridgman furnace for crystal growth. An “oscillating” temperature profile was first applied to melt the sample thoroughly. When the ampoule was placed in the hot zone, the temperature in the hot zone was set to 1208(5) K for 24 hours, then cooled down to 973(5) K for 12 hours and afterwards the temperature was increased to 1123(5) K for 120 hours. After the melt process, the ampoule was dropped at a rate of 2.3 mm h^–1^ from the hot zone to the cold zone, where the temperature in the hot zone was 1123(5) K, and the temperature of the cold zone was 973(5) K. The crystal growth was completed in five days. Several crystals were grown to check for reproducibility. The Archimedean densities of the crystal slices vary in the range of 99.4–99.8% from theoretical X-ray density.

### Elemental analysis

Elemental analysis of selected crystals was carried out on a Hitachi S4100T scanning electron microscope (SEM) with energy-dispersive X-ray (EDX) microanalysis (Oxford INCA Energy) to check for a consistent elemental ratio of the elements in the samples (Fig. S3 and Table S3†).

### Single-crystal X-ray diffraction

Single-crystal diffraction experiments were collected at 90 K using a Bruker AXS SMART diffractometer with an APEX-II CCD detector with Mo-K_α_ radiation. The datasets were recorded as ω-scans with a 0.4° step width and integrated with the Bruker SAINT software package.^27^ Multi-scan absorption corrections were applied.^27^ The solution and refinement of the crystal structure were carried out using the SHELX-2014 suite of programs.^28^ The final refinements were performed using anisotropic atomic displacement parameters for all atoms. A summary of pertinent information relating to unit cell parameters, data collection, and refinements is provided in Table 1 and the atomic parameters and interatomic distances are provided in Tables S1 and S2.† Further details of the crystal structure determination may be obtained from Fachinformationszentrum Karlsruhe, Germany, by quoting the depository number CSD-433052.

**Table 1:** Selected single crystal data and structure refinement parameters for Ba_8_Cu_14_Ge_6_P_26_

Temperature	90(2) K
Radiation, wavelength	Mo-K_α_, 0.71073 Å
Space group	*Pm*3*n* (no. 223)
Unit cell dimensions	*a* = 10.0626(8) Å
Unit cell volume, *Z*	1018.9(2) Å^3^, 1
Density (calc.)	5.26 g cm^–3^
Absorption coefficient	19.61 cm^–1^
Data/parameters	333/25
Goodness-of-fit	1.14
Final *R* indices [*I* > 2*σ*(*I*)]	*R*_1_ = 0.015
	w*R*_2_ = 0.025
Final *R* indices [all data]	*R*_1_ = 0.017
	w*R*_2_ = 0.026
Max diff. peak and hole	0.80 and –0.53

### Transmission electron microscopy (TEM)

Samples for electron microscopy were ground under ethanol, and the resulting dispersion was transferred to a holey carbon film fixed on a 3 mm copper grid. Electron diffraction (ED) studies were performed using a Tecnai G2 30 UT (LaB_6_) microscope operated at 300 kV with 0.17 nm point resolution. High-angle annular dark field (HAADF)-scanning TEM (STEM) studies and EDX elemental mapping were performed using a JEM ARM200F cold FEG double aberration corrected electron microscope operated at 200 kV and equipped with a large solid-angle CENTURIO EDX detector and Quantum EELS spectrometer.

### X-ray and neutron powder diffraction and PDF

All samples were characterized by X-ray powder diffraction (XRD) using a Rigaku Miniflex 600 diffractometer employing Cu-K_α_ radiation. High-resolution room temperature synchrotron powder XRD data were collected at beamline 11-BM (*λ* = 0.459266 Å) at the Advanced Photon Source (APS) at Argonne National Laboratory (ANL) (Fig. 1). The refined unit cell parameter of Ba_8_Cu_14_Ge_6_P_26_ from synchrotron X-ray diffraction data at room temperature, *a* = 10.09454(1) Å, agrees with the result obtained from single-crystal refinement, *a* = 10.0626(8) Å at 90 K. Neutron powder diffraction time-of-flight data for neutron pair distribution function (N-PDF) were collected at the NOMAD beamline at the Spallation Neutron Source (SNS) at Oak Ridge National Laboratory (ORNL). Additional high-energy total X-ray scattering (X-PDF) data (*λ* = 0.21 Å) were collected at the PDF beamline 11-ID-B at APS ANL. Neutron and X-ray PDF data were analyzed using PDFGUI.^29^,^30^

### Transport properties

Transport properties were studied on several slices of different Bridgman-grown crystals. Low-temperature transport properties in the temperature range of 2–400 K were studied using the commercial multipurpose Physical Properties Measurement System (PPMS, Quantum Design). The Seebeck thermopower and thermal conductivity were measured using the thermal transport option. The electrical resistivity was measured by a standard four-point alternating-current technique to exclude the resistance of the leads. High-temperature thermal conductivity was carried on a Netzsch LFA 457. The Seebeck coefficient and resistivity measurements were conducted simultaneously on a Linseis LSR-3/1100 instrument system under helium atmosphere. The high-temperature data were additionally verified at the Jet Propulsion Laboratory using both custom and commercial apparatus. The Seebeck coefficient was measured using the light-pipe method with tungsten–niobium thermocouples under high vacuum in a custom set-up.^31^ Temperature-dependent resistivity was measured using a Van der Pauw 4-point method with tungsten pressure contact probes.^32^ Thermal diffusivity was measured using a Netzsch LFA 404 system. The temperature was limited to 800 K to prevent sublimation of the samples during measurements. All transport measurements were taken during both heating and cooling (heating rate of 180 K h^–1^), and showed no hysteresis. The combined measurement uncertainty in the thermoelectric figure of merit is generally assumed to be ∼20%.

## Results and discussion

### Composition and crystal structure

Our motivation to search for new clathrates was to produce electron-balanced compounds with the semiconductor-like properties that are highly desirable for thermoelectric applications. The clathrate Ba_8_Cu_16_P_30_ was reported to exhibit metallic properties due to an insufficient number of valence electrons to form four bonds per clathrate framework atom.^25^,^33^,^34^ In this work we realized an electron-balanced compound by the replacement of some Cu (1 valence electron) and P (5 valence electrons) atoms with Ge (4 valence electrons) atoms. Several synthesized samples with different nominal Ba/Ge/Cu/P ratios contained the same phase with a similar composition as was evidenced by EDX and unit cell parameter determinations. The precise elemental ratio in the new clathrate was determined by a combination of three different methods. First, the refinement of single-crystal X-ray diffraction data resulted in the chemical formula of Ba_8_M_20.1(2)_P_25.9(2)_, where M indicates the merging of Cu and Ge elements due to their similar X-ray atomic scattering factors. Tests of different crystals selected from different batches all resulted in an identical composition within the standard deviations of the refinements. Afterwards, the chemical composition of different crystals analyzed by EDX spectroscopy resulted in the formula Ba_8_Cu_14.4(2)_Ge_5.5(1)_P_22(1)_ when normalized to 8 Ba atoms (Table S3†). Note that light P atoms cannot be accurately determined by EDX, and some overlap of Cu and Ge lines hinder precise determination of the elemental contents. However, the averaged amount of Cu + Ge from EDX is 19.9(3), which agrees very well with the results from single-crystal diffraction. Finally, Zintl counting exactly predicts the Ba_8_Cu_14_Ge_6_P_26_ composition for 20 metal Cu + Ge atoms. Indeed, in the clathrate-I system Ba_8_X_46_, where X is the framework atom, each Ba atoms donates 2 electrons to the framework becoming Ba^2+^, and 4 electrons are required for per framework atom, X, to achieve an electron balance by forming 4 covalent bonds in the framework.^17^ Since there are 46 atoms in the framework, a total of 46 × 4 = 184 electrons are required. Besides the 16 electrons from Ba atoms, the framework atoms need additional 168 electrons coming from X elements. Assuming the total Cu + Ge content is equal to 20 and 26 P atoms in the framework, the only electron-balanced solution is 14 Cu atoms (14 × 1 = 14 electrons), 6 Ge atoms (6 × 4 = 24 electrons), and 26 P atoms (26 × 5 = 130 electrons), resulting in exactly 168 electrons. The electron-balanced nature of Ba_8_Cu_14_Ge_6_P_26_ was further confirmed by properties measurements (*vide infra*). All our attempts to synthesize samples with Ba_8_Cu_14+*x*_Ge_6–*x*_P_26_ nominal compositions, where *x* varies from 0.1 to 1, resulted in no detectable unit cell parameter shifts or changes in transport properties, indicating that Ba_8_Cu_14_Ge_6_P_26_ is a line compound.

Ba_8_Cu_14_Ge_6_P_26_ crystallizes in the clathrate-I structure type with the space group *Pm*3*n* (no. 223). Clathrate-I is the most abundant structure type among inorganic clathrates. Its crystal structure can be described as composed of two types of polyhedra: pentagonal dodecahedra and tetrakaidecahedra. The Ba atoms are encapsulated inside both types of polyhedra. Tetrakaidecahedra share their hexagonal faces, forming columns running along all three main crystallographic directions and the residual space filled by dodecahedra (Fig. 1 inset). There is no evidence for vacancies on the Ba sites. Three framework sites, 6c, 16i, and 24k are jointly occupied by Cu, Ge and P. Cu and Ge, atomic numbers 29 and 32, are difficult to distinguish by X-ray diffraction due to similar atomic scattering factors. During the refinement, all framework sites were originally refined as jointly occupied by Cu and P under the constraints of equivalent atomic displacement parameters and the total occupancy of each site was fixed to 100%. Similar refinements with all framework positions set as Ge/P resulted in a slightly lower but similar M/P ratio. At the final stages of the refinement, each framework site was refined as jointly occupied by Cu + Ge + P. An additional compositional restraint was applied to fix the total elemental content in the structure to 14Cu + 6Ge + 26P (Tables 1, S1, and S2†). Mixed occupancy of the framework positions by several different elements, including Cu/Ge mixing, is common for the tetrel clathrates, such as Ba_8_Ga_16_Ge_30_,^35^ Ba_8_Ga_17.1_Ge_25.6_Sb_2.7_,^36^ and Ba_8_Cu_6–*x*_Ge_40+*x*_.^37^

Superstructures and partial orderings are well-known in the crystal chemistry of clathrates. For the type-I clathrate, the aristotype structure crystallizes in space group *Pm*3*n* (no. 223) with *a* ≈ 10 Å and volume *V* ≈ 1000 Å^3^. Different kinds of superstructures have been reported for type-I clathrates ranging from lowered symmetry within the same volume unit cell^38^ to an increase of the unit cell volume by a factor of 4–8 due to either the ordering of vacancies in the tetrel-based clathrates, as in the cases of Cs_8_Sn_44_ or Ba_8_Ge_43_, or the separation of atoms of different chemical natures over distinct framework sites, as in the case of Ba_8_Cu_16_P_30_ and Ba_8_Au_16_P_30_.^15^,^24^,^39^–^41^ The vacancies in the framework or guest positions do not necessarily lead to the formation of a superstructure.^42^,^43^ Alternatively, the formation of short-range ordering without a long-range superstructure is possible, but is more difficult to detect with diffraction techniques and requires the application of local probes. For example, the recently reported type-I clathrate Ba_8_M_16+*y*_P_30–*y*_ (M = Cu, Zn) exhibits partial superstructural ordering confirmed with ED, STEM, and PDF, and can also be detected due to presence of weak extra diffraction peaks in its synchrotron powder diffraction pattern.^25^ No such extra peaks were detected in the diffraction pattern of Ba_8_Cu_14_Ge_6_P_26_ sample (Fig. 1 inset), which demonstrates the absence of long-range superstructural ordering in Ba_8_Cu_14_Ge_6_P_26_. To further investigate possible short-range ordering, we applied electron microscopy.

**Fig. 2:**
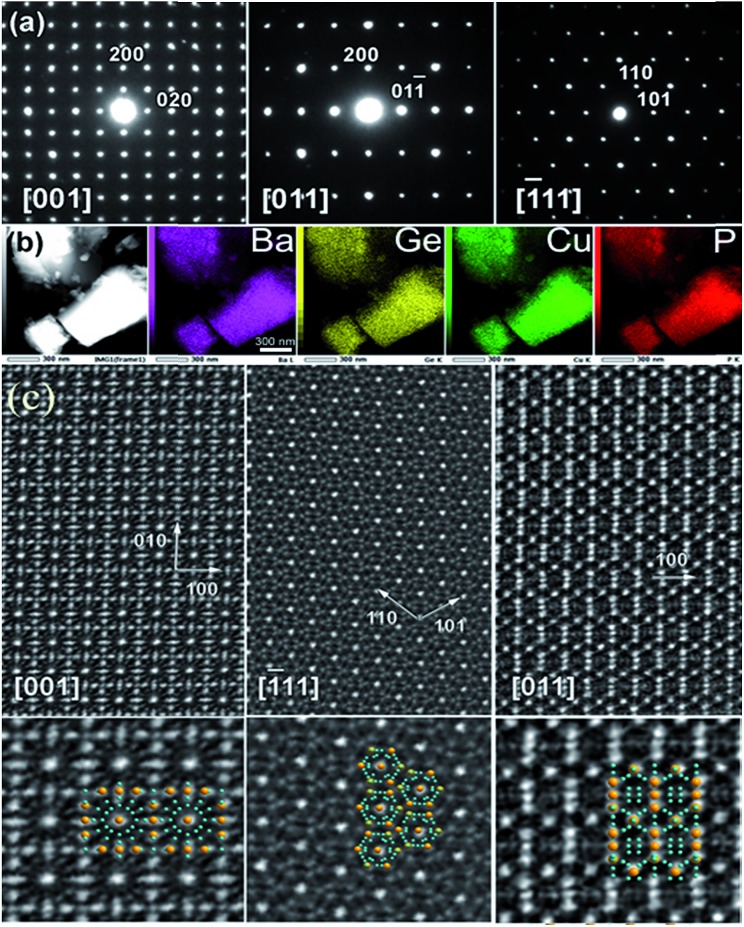
(a) ED patterns along the main cubic zones and (b) elemental mapping of selected Ba_8_Cu_14_Ge_6_P_26_ crystals. Ba: purple, Ge: yellow, Cu: green, P: red. (c) HAADF-STEM images along main zone axes [001], [111] and [011] of Ba_8_Cu_14_Ge_6_P_26_ are shown. The insets in the bottom show structural fragment overlaps (Ba: yellow, Cu/Ge/P: blue).

Elemental mapping shows uniform distributions of the Ba, Cu, Ge, and P elements over the entire studied crystals (Fig. 2b). All observed diffraction spots in the ED patterns can be indexed in the cubic clathrate-I unit cell. No superstructural reflections were found (Fig. 2a). This is in a stark contrast to the Ba_8_M_16+*y*_P_30–*y*_ (M = Cu, Zn) clathrate where preferential Cu–Zn bonding resulted in the formation of a trigonal superstructure, detectable by ED.^25^ High-resolution HAADF-STEM investigations confirm the well-ordered cubic nature of the Ba_8_Cu_14_Ge_6_P_26_ crystallites with good agreement to the overlayed structural models (Fig. 2c). No structural defects, like antiphase boundaries or stacking faults, were observed in the studied Ba_8_Cu_14_Ge_6_P_26_ crystallites.

**Fig. 3:**
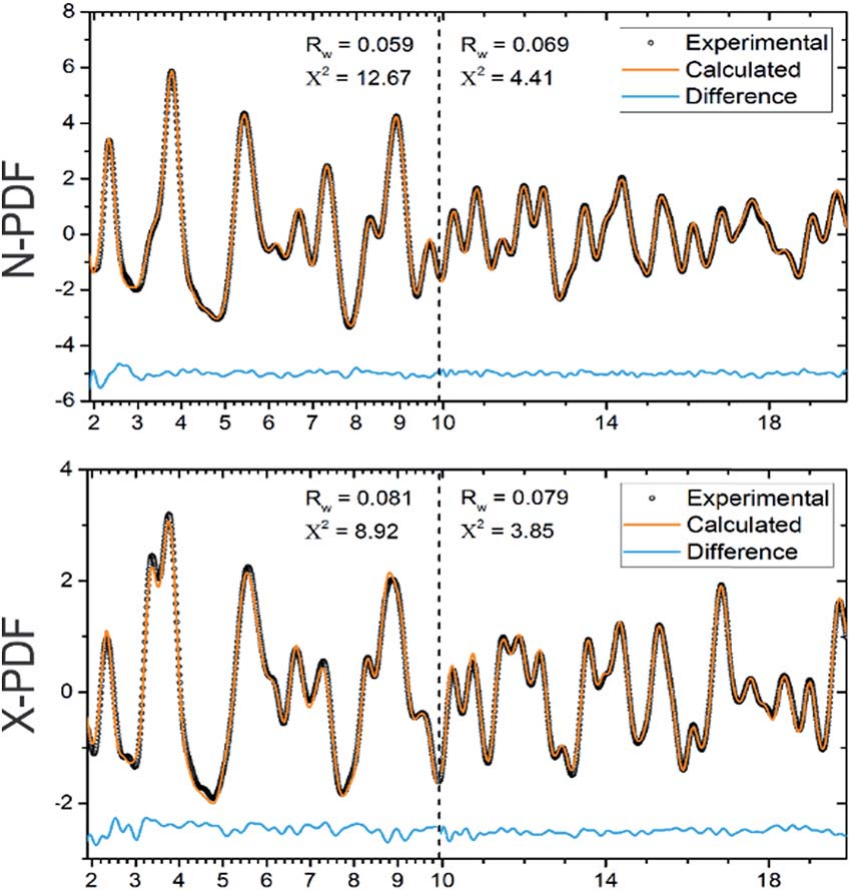
Experimental neutron (top) and X-ray (bottom) pair distribution functions and their fits are shown for a cubic model of Ba_8_Cu_14_Ge_6_P_26_. Experimental data are open black circles, calculated fits are red lines, and difference curves are shown as blue lines.

Synchrotron X-ray and neutron PDF analyses also confirmed the absence of local ordering in Ba_8_Cu_14_Ge_6_P_26_ (Fig. 3). Independent fittings of the short-range (1.9 Å ≤ *r* ≤ 9.9 Å) and long range (9.9 Å ≤ *r* ≤ 19.9 Å) PDF regions were performed using the cubic *Pm*3*n* model. Due to the similarity of the neutron scattering lengths for Cu (7.7 fm) and Ge (8.2 fm) the occupancies of the atomic positions in the framework were fixed to the values obtained from the single crystal X-ray diffraction experiment and were not refined further. The structural model determined from the single crystal X-ray experiment describes the PDF patterns well, indicating the absence of any short-range ordering. The presence of local ordering in the clathrate framework results in significant perturbations of the short-range PDF data.^25^

Based on TEM, synchrotron XRD, and PDF results, the crystal structure of Ba_8_Cu_14_Ge_6_P_26_ is established to have primitive cubic *Pm*3*n* symmetry with a uniform distribution of the framework elements and without local ordering, superstructure, or defects. The main difference between the Cu–Zn–P and Cu–Ge–P frameworks is the preference for Cu and Zn to form chemical bonds to each other. We have elegantly shown that an increase of the Zn content resulted in the formation of a clathrate where all metal atoms are joined in Cu–Zn dumbbells.^26^ Apparently, such bonding preference is absent in the case of the Ba–Cu–Ge–P clathrate, which resulted in the formation of the cubic structure with a uniform distribution of Cu, Ge, and P over the clathrate framework sites.

Although clathrate-I is a well-known structure type, Ba_8_Cu_14_Ge_6_P_26_ is the first representative of inorganic clathrates with electropositive guest atoms where the framework is composed of a transition metal, tetrel element, and pnicogen. The tetrel-based clathrates are a prevailing group with over 100 compounds with diverse properties.^17^ Until now, only a dozen tetrel-free clathrates have been reported, which include phosphides, Ba_8_M_16_P_30_ (ref. 15 and 24) and AeM_2_P_4_ (Ae = Sr, Ba; M = Ni, Cu),^18^ arsenides and antimonides, A_8_E_18_Pn_28_, A = Rb, Cs; E = Zn, Cd; Pn = As, Sb.^44^,^45^ Prior to our work, there was an insurmountable gap between these two clathrate groups, despite the reported attempts to dope Sb into tetrel clathrates.^36^ Ba_8_Cu_14_Ge_6_P_26_ fills the gap between tetrel-based and pnicogen-based clathrates and provides new insight and opportunities for clathrate research. High thermal stability and the electron-balanced nature of this clathrate result in outstanding transport properties.

### Thermal stability

**Fig. 4:**
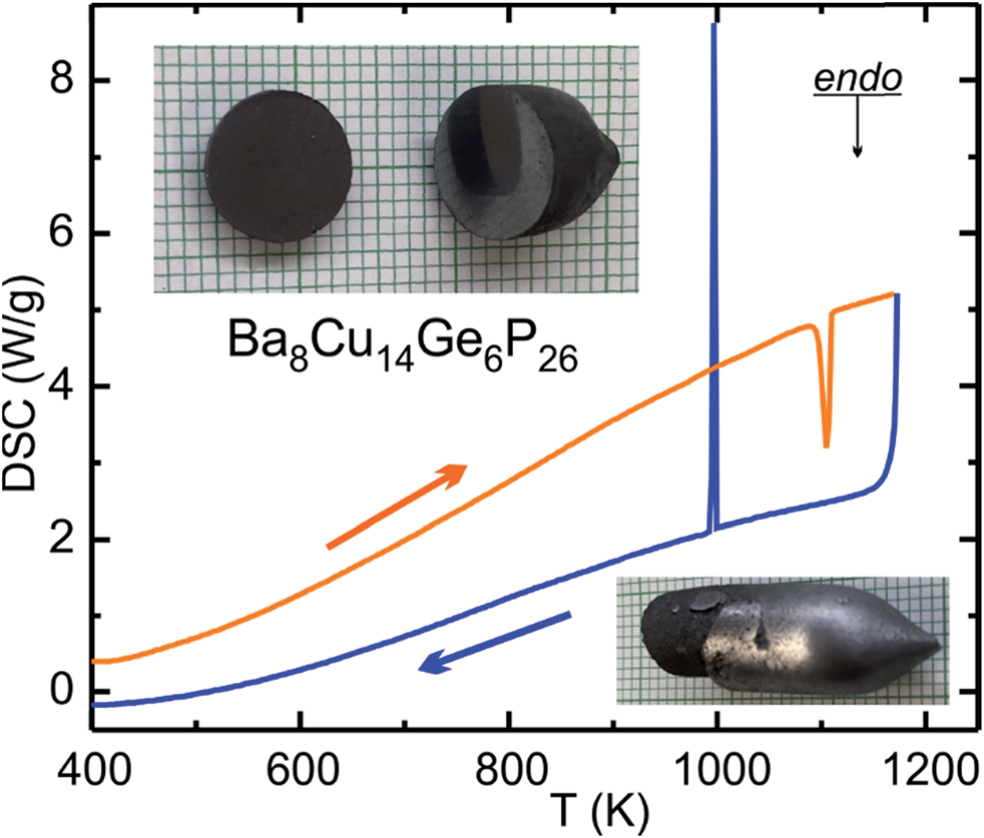
DSC results for Ba_8_Cu_14_Ge_6_P_26_. Heating: orange curve; cooling: blue curve. The inset are photographs of (bottom) Bridgman growth crystal and (top) slices of such crystals with mirror-like surface on a background of mm-grid paper.

DSC characterization results for Ba_8_Cu_14_Ge_6_P_26_ are shown in Fig. 4 and S4.† Ba_8_Cu_14_Ge_6_P_26_ melts congruently at 1108(3) K and recrystallizes at 1006(3) K. No decomposition or phase transition were detected upon melting and crystallization, which was verified by powder XRD (Fig. S2†). The thermal stability of Ba_8_Cu_14_Ge_6_P_26_ was further confirmed by powder XRD of the phase after annealing a single-phase sample in a sealed tube at 1223 K for 12 hours. The congruent melting of Ba_8_Cu_14_Ge_6_P_26_ makes the single-crystal growth process feasible. We applied the vertical Bridgman method to grow large crystals of Ba_8_Cu_14_Ge_6_P_26_ (Fig. 4 bottom inset). A slice of the grown crystal with a mirror-like surface is also shown in Fig. 4 top inset.

### Thermoelectric properties

**Fig. 5:**
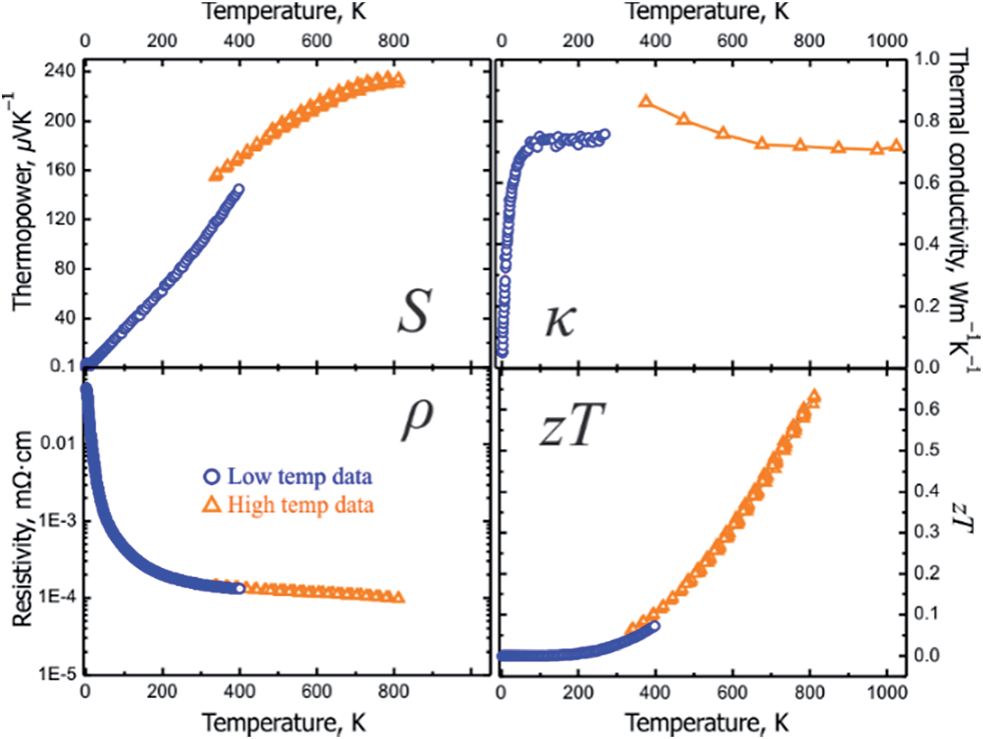
Low- (blue) and high-temperature (orange) transport properties on a slice of the Bridgman growth crystal of Ba_8_Cu_14_Ge_6_P_26_: (top left) Seebeck thermopower; (top right) thermal conductivity; (bottom left) electrical resistivity; (bottom right) thermoelectric figure of merit, *ZT*. The estimated standard deviations for *ZT* are ∼20%, see Fig. S6† and discussion therein.

The presence of grain boundaries may mask the intrinsic properties of solids. In thermoelectric characterization of polycrystalline materials, the effect of grain boundaries is minimized by studying dense pellets. However, large crystals are more suitable for studying the intrinsic properties of compounds. For Ba_8_Cu_14_Ge_6_P_26_, characterization of slices from several crystals grown by the vertical Bridgman method were performed (Fig. 4 inset). The data obtained at UC Davis were verified by measurements at JPL. Note that JPL measurements were run on a slice of a different crystal and using different equipment (Fig. S6†). Both the low-temperature and high-temperature thermoelectric properties show good agreement with each other with small discontinuities at 300–400 K due to the different methods and instruments used (Fig. 5).

The thermopower for Ba_8_Cu_14_Ge_6_P_26_ samples is positive in the whole studied temperature range (10–812 K) indicating that holes are the main charge carrier type. The Seebeck thermopower was measured several times during heating and cooling in the high-temperature range with good consistency. At room temperature, a promising value for thermopower, 102 μV K^–1^, was achieved. At the maximum measured temperature, 812 K, the thermopower value increases up to 234 μV K^–1^ (Table 2 and Fig. 5).

**Table 2:** A summary of the thermoelectric properties of Ba_8_Cu_14_Ge_6_P_26_ and related clathrates

	At 300 K				At 812 K				Ref.
	*S* μV K^–1^	*ρ* μΩ m	*κ* W m^–1^ K^–1^	*ZT*	*S* μV K^–1^	*ρ* μΩ m	*κ* W m^–1^ K^–1^	*ZT*	
Ba_8_Cu_14_Ge_6_P_26_	101.5	149.7	0.77	0.03	234	98	0.7	0.63	This work
Ba_8_Cu_5.3_Ge_39.8_	154	1610	1.4	0.003	—	—	—	—	46
Ba_8_Ga_15.8_Al_3_Ge_27_^*a*^	187	83	1.05	0.12	254^*b*^	78^*b*^	1.0^*b*^	0.6^*b*^	47
Ba_8_Cu_16_P_30_	12.8	11.7	1.2	0.004	46	19	1.4	0.07	This work

^*a*^Ba_8.01_Ga_15.79_Al_2.95_Ge_26.91_ is a p-type Ge-based clathrate with high thermoelectric performance.

^*b*^Data at peak *ZT* temperature of 750 K.

Ba_8_Cu_14_Ge_6_P_26_ is the first quaternary anionic clathrate connecting tetrel-based and tetrel-free clathrate families. It is reasonable to compare the properties of Ba_8_Cu_14_Ge_6_P_26_ to the properties of its ternary counterparts, Ba_8_Cu_5.3_Ge_39.8_ (ref. 46) and Ba_8_Cu_16_P_30_, which are also p-type conductors. Additionally, the performance of Ba_8_Cu_14_Ge_6_P_26_ is compared to Ba_8_Ga_15.8_Al_3_Ge_27_, the one of the best performing Ge-based, p-type thermoelectric clathrate (Table 2).^47^ For Ba_8_Cu_16_P_30_, no high-temperature thermoelectric properties have been reported,^33^ so we re-synthesized this sample and measured high-temperature thermoelectric properties shown in the last line of Table 2. The Seebeck thermopower value at 300 K of Ba_8_Cu_14_Ge_6_P_26_, 102 μV K^–1^, is much higher than that for Ba_8_Cu_16_P_30_ (13 μV K^–1^) and lower than the thermopowers for Ba_8_Cu_5.3_Ge_39.8_ (154 μV K^–1^) and Ba_8_Ga_15.8_Al_3_Ge_27_ (187 μV K^–1^). With increasing temperature, the Seebeck value of Ba_8_Cu_14_Ge_6_P_26_, 234 μV K^–1^ at 812 K, is comparable to the thermopower of the Ba_8_Ga_15.8_Al_3_Ge_27_ clathrate, 254 μV K^–1^ at 750 K.

The resistivities of multiple single-crystalline and pressed polycrystalline Ba_8_Cu_14_Ge_6_P_26_ samples decrease with increasing temperature over the whole temperature range, which indicates a thermally activated behavior typical for semiconductors (Fig. 5, bottom left). The resistivity of Ba_8_Cu_14_Ge_6_P_26_ at 300 K is 150 μΩ m, which decreases to 98 μΩ m at 812 K. Ba_8_Cu_16_P_30_ exhibits lower resistivity, 12 μΩ m, at 300 K. In turn, the room temperature resistivity for Ba_8_Cu_5.3_Ge_39.8_ is much higher, 1610 μΩ m. At 812 K, the resistivity of Ba_8_Cu_14_Ge_6_P_26_ remains the highest compared to the resistivities of Ba_8_Ga_15.8_Al_3_Ge_27_ and Ba_8_Cu_16_P_30_ (Table 2). The resulting power factor for Ba_8_Cu_14_Ge_6_P_26_ at 812 K is 5.62 μW cm^–1^ K^–2^. The relatively high resistivity of Ba_8_Cu_14_Ge_6_P_26_ indicates that further optimization of its thermoelectric efficiency can be performed *via* tuning the electrical conductivity by charge carrier concentration modifications. For example, for Ba_8_Ga_16+*x*_Ge_30–*x*_ p-type clathrates, the typical values of *ZT* are less than 0.5.^48^ However, a significantly higher performance was recently reported for a Bridgman growth sample with the composition Ba_8_Ga_16.6_Ge_28.7_.^49^ While the Seebeck coefficient and thermal conductivity for this clathrate are comparable to those of Ba_8_Cu_14_Ge_6_P_26_, adjustment of the Ga/Ge ratio resulted in the significant reduction of resistivity down to 35 μΩ m at 823 K.^49^ Investigations of aliovalent doping into the cation sublattice of Ba_8_Cu_14_Ge_6_P_26_ are currently underway.

Ba_8_Cu_14_Ge_6_P_26_ inherits the low thermal conductivity of the clathrate family of structures due to its intrinsically complex host–guest structure and rattling of guest atoms.^10^,^16^,^17^ The thermal conductivity of Ba_8_Cu_14_Ge_6_P_26_ increases with temperature and reaches a plateau at 100 K of 0.75 W m^–1^ K^–1^ without a pronounced maximum in the 50–150 K range, which is not typical for crystalline solids and more common for amorphous materials. Such behavior was observed for some clathrates with complex disorder in the framework.^16^,^50^,^51^ The statistical disorder of Cu, Ge, and P seems to be responsible for such behavior and the extremely low values of the thermal conductivity for Ba_8_Cu_14_Ge_6_P_26_. As temperature increases, the thermal conductivity decreases down to a value of 0.72 W m^–1^ K^–1^ at 812 K. The thermal conductivity value of Ba_8_Cu_14_Ge_6_P_26_ is lower than the thermal conductivities of a majority of clathrate compounds (1–3 W m^–1^ K^–1^).^16^,^17^ The thermal conductivity of Ba_8_Cu_14_Ge_6_P_26_ is also comparable to that of some of the state-of-art thermoelectric materials, such as Yb_14_MnSb_11_ (0.7 W m^–1^ K^–1^ at 300 K),^52^ Bi_0.5_Sb_1.5_Te_3_ (0.7 W m^–1^ K^–1^ at 300 K),^53^ [010] direction of the SnSe single crystal (0.7 W m^–1^ K^–1^ at 300 K),^54^ and Ca_3_AlSb_3_ (1.3 W m^–1^ K^–1^ at 300 K).^55^,^56^ The thermal conductivity consists of a charge carrier contribution and phonon contribution, *κ*_total_ = *κ*_e_ + *κ*_L_ = *LT*/*ρ* + *κ*_L_ (*κ*_e_: electronic thermal conductivity; *κ*_L_: lattice thermal conductivity; *L*: Lorenz number; *ρ*: electrical resistivity; *T*: absolute temperature), where the *L* can be estimated based on the Seebeck coefficient values.^57^ The lattice and electronic contributions to total thermal conductivity for Ba_8_Cu_14_Ge_6_P_26_ are shown in Fig. S5.† The lattice thermal conductivity exhibits a similar trend to the total thermal conductivity and reaches 0.58 W m^–1^ K^–1^ at 812 K, which is comparable to the lattice thermal conductivity at 800 K for many state-of-the-art thermoelectric materials, such as Yb_14_MnSb_11_ (0.51 W m^–1^ K^–1^),^58^ Sr_3_GaSb_3_ (0.52 W m^–1^ K^–1^),^59^ and Ba_8_Ga_16_Ge_30_ (0.65 W m^–1^ K^–1^).^60^

Finally, due to a combination of the high thermopower, ultralow thermal conductivity, and intermediate electrical conductivity, the Bridgman growth sample of Ba_8_Cu_14_Ge_6_P_26_ exhibits a promising thermoelectric figure of merit, *ZT*, of 0.63 at 812 K (Fig. 5, bottom right). The average *ZT* for the 400–800 K range is 0.35. The maximum *ZT* of Ba_8_Cu_14_Ge_6_P_26_ at 812 K is 9 times higher than the value for the ternary tetrel-free counterpart, Ba_8_Cu_16_P_30_ (0.07). Ba_8_Cu_14_Ge_6_P_26_ is among the most-efficient p-type Ge-containing clathrate thermoelectrics. Compared to the highly tuned and more expensive Ba_8_Ga_15.8_Al_3_Ge_27_ compound, the initial achievement of *ZT* = 0.63 makes Ba_8_Cu_14_Ge_6_P_26_ an attractive platform for the development of mid- and high-temperature thermoelectrics. Ba_8_Cu_14_Ge_6_P_26_ exhibits comparable thermoelectric efficiency to the unoptimized Yb_14_MnSb_11_, which is the base for the development of the most efficient high-temperature p-type thermoelectric materials.^52^,^61^–^64^ For example, at 812 K the thermoelectric efficiency for Ba_8_Cu_14_Ge_6_P_26_ and pristine Yb_14_MnSb_11_ are 0.63 and 0.53, respectively. Many strategies to tune carrier concentration and enhance electrical conductivity, such as doping,^5^ band engineering,^65^ and aliovalent substitutions in the framework and guest sublattices^16^,^17^ may be applied to improve the thermoelectric efficiency of Ba_8_Cu_14_Ge_6_P_26_. From a chemistry point-of-view, a novel clathrate system connecting tetrel-based clathrates and tetrel-free clathrates may reinvigorate interest in searching for new clathrates with high thermoelectric performance.

## Conclusions

A new clathrate compound, Ba_8_Cu_14_Ge_6_P_26_, was synthesized by a traditional solid-state reaction and grown as single crystal *via* the vertical Bridgman growth method. Ba_8_Cu_14_Ge_6_P_26_ crystallizes in a type-I clathrate structure with cubic system *Pm*3*n* space group. The three-dimensional framework is built from uniformly distributed Cu, Ge and P atoms, which is confirmed by ED, STEM, single crystal and powder synchrotron diffraction, and X-ray and neutron PDF analyses. The congruent melting of Ba_8_Cu_14_Ge_6_P_26_, as revealed by DSC, allows for crystal growth *via* vertical Bridgman growth, which is beneficial for exploring intrinsic physical properties. Thermoelectric characterizations show that Ba_8_Cu_14_Ge_6_P_26_ is a p-type semiconductor with a promising figure of merit, *ZT* = 0.63 at 812 K. Ba_8_Cu_14_Ge_6_P_26_ exhibits auspicious thermoelectric properties outperforming majority of p-type Ge-based and tetrel-free clathrates. The efforts to reduce the resistivity of Ba_8_Cu_14_Ge_6_P_26_ through aliovalent doping are currently underway.

## Conflicts of interest

There are no conflicts to declare.

## Author contributions

All authors have given approval to the final version of the manuscript.

## Funding sources

This research was supported by the U.S. Department of Energy, Office of Basic Energy Sciences, Division of Materials Science and Engineering under Award DE-SC0008931. Part of this work was performed at the California Institute of Technology/Jet Propulsion Laboratory under contract with the National Aeronautics and Space Administration. This part of the work was supported by the NASA Science Missions Directorate's Radioisotope Power Systems Thermoelectric Technology Development Project. Use of the Advanced Photon Source at Argonne National Laboratory was supported by the U.S. Department of Energy, Office of Science, Office of Basic Energy Sciences, under Contract No. DE-AC02-06CH11357. Research conducted at ORNL's Spallation Neutron Source was sponsored by the Scientific User Facilities Division, Office of Basic Energy Sciences, U.S. Department of Energy.

## Supplementary Material

Supplementary information

Crystal structure data
