# Supplementary material for: High-efficiency thermoelectric Ba8Cu14Ge6P26: bridging the gap between tetrel-based and tetrel-free clathrates
Source: Chem Sci. 2017 Sep 29;8(12):8030–8. doi: 10.1039/c7sc03482b (PMC5853772; doi:10.1039/c7sc03482b)
Supplement: Supplementary file 1 [file SC-008-C7SC03482B-s001.pdf]

Electronic Supplementary Material (ESI) for Chemical Science.
This journal is © The Royal Society of Chemistry 2017

High-efficiency Thermoelectric $Ba_8Cu_{14}Ge_6P_{26}$: Bridging the gap between tetrel-based and tetrel-free clathrates

Jian Wang,[1] Oleg I. Lebedev,[2] Kathleen Lee,[1,3] Juli-Anna Dolyniuk,[1] Peter Klavins,[4] Sabah Bux,[3] Kirill Kovnir[1,5,6*]

Supporting information

Page S1. **Figure S1.** Calculated and experimental lab powder XRD of $Ba_8Cu_{14}Ge_6P_{26}$.

Page S2. **Figure S2.** Powder X-ray diffraction patterns for the as-synthesized $Ba_8Cu_{14}Ge_6P_{26}$ sample and the sample after DSC experiment.

Page S3. **Table S1.** Refined atomic coordinates and equivalent atomic displacement parameters for $Ba_8Cu_{14}Ge_6P_{26}$.

Page S4. **Table S2.** Selected interatomic distances for $Ba_8Cu_{14}Ge_6P_{26}$.

Page S5. **Table S3**. EDS results for selected $Ba_8Cu_{14}Ge_6P_{26}$ crystals.

Page S6. **Figure S3**. SEM images of selected $Ba_8Cu_{14}Ge_6P_{26}$ crystal.

Page S7. **Figure S4.** DSC results of three cycles of heating and cooling of the same $Ba_8Cu_{14}Ge_6P_{26}$ sample.

Page S8. **Figure S5.** Thermal conductiviy of $Ba_8Cu_{14}Ge_6P_{26}$ with electronic and lattice contributions to total thermal conductivity.

Page S9. **Figure S6.** Comparison of thermoelectric efficiency of $Ba_8Cu_{14}Ge_6P_{26}$ for three different samples measured at UC Davis and JPL.

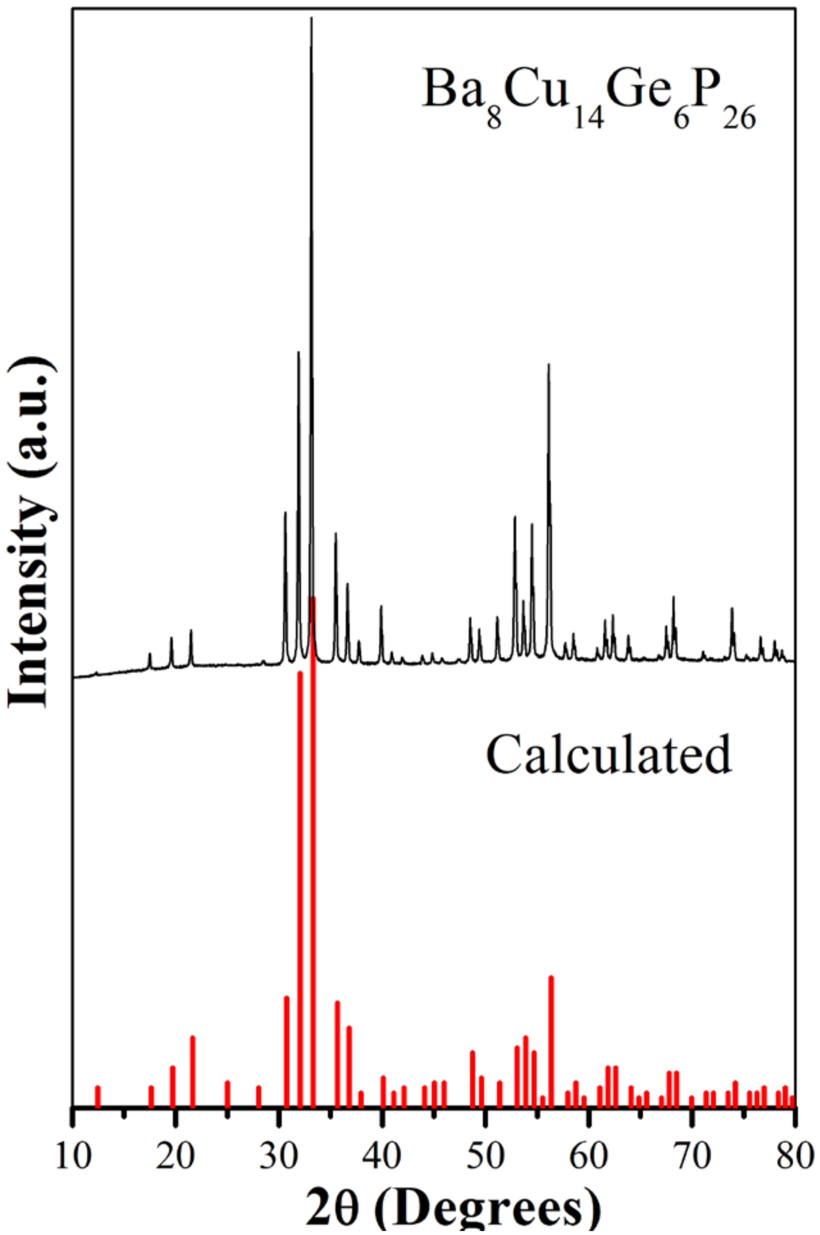

Figure S1. Calculated and experimental lab powder X-ray diffraction patterns of $Ba_8Cu_{14}Ge_6P_{26}$.

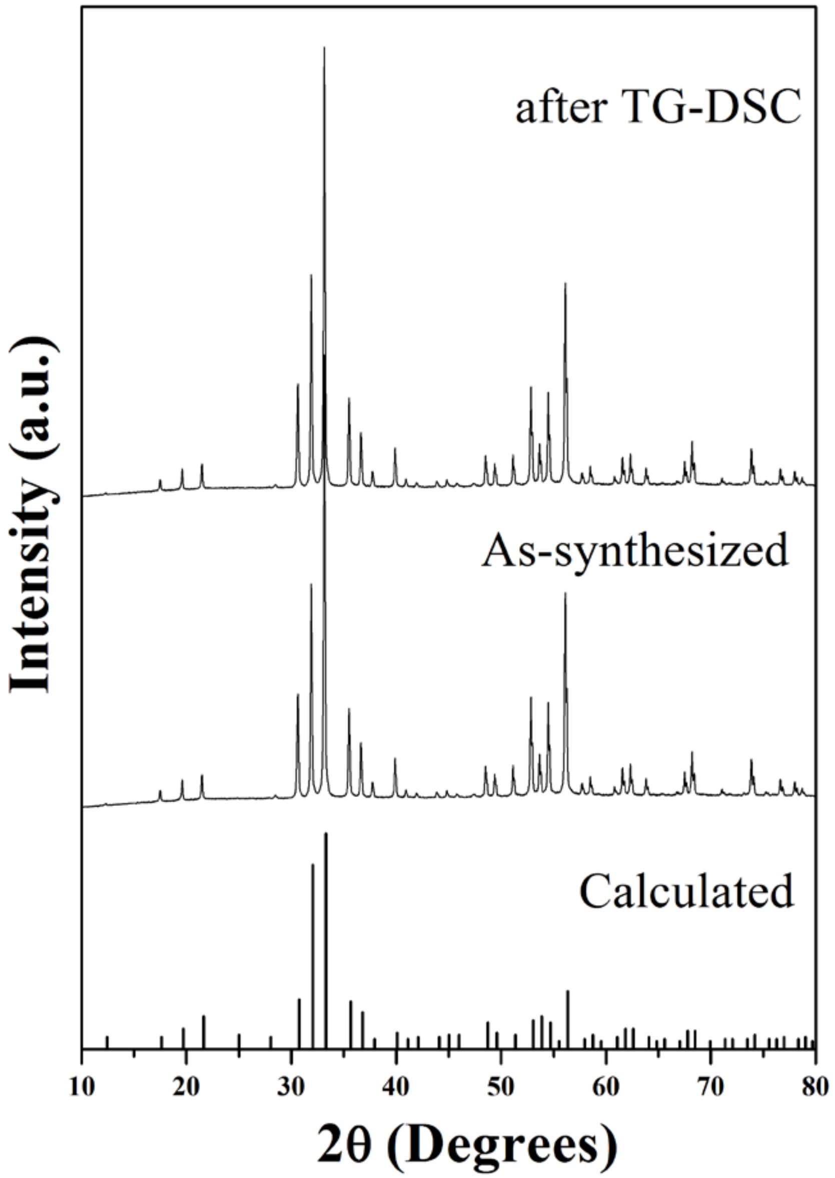

Figure S2. Powder X-ray diffraction patterns of for the as-synthesized $Ba_8Cu_{14}Ge_6P_{26}$ sample and the sample after DSC experiment.

Table S1. Refined atomic coordinates and equivalent atomic displacement parameters for $Ba_8Cu_{14}Ge_6P_{26}$.

Atom	Wyckoff	*x/a*	*y/b*	*z/c*	*S.O.F.*	U_{eq} (Å^2) $^{a)}$
			$Ba_8Cu_{14}Ge_6P_{26}$			
Ba1	2*a*	0	0	0	1	0.0061(1)
Ba2	6*d*	¼	½	0	1	0.0214(1)
P1	6*c*	¼	0	½	0.61(3)	0.0066(4)
Cu1	6*c*	¼	0	½	0.35(3)	0.0066(4)
Ge1	6*c*	¼	0	½	0.04(3)	0.0066(4)
P2	16*i*	0.18502(4)	*x*	*x*	0.69(1)	0.0075(2)
Cu2	16*i*	0.18502(4)	*x*	*x*	0.22(1)	0.0075(2)
Ge2	16*i*	0.18502(4)	*x*	*x*	0.09(1)	0.0075(2)
P3	24*k*	0	0.30860(4)	0.12068(4)	0.47(1)	0.0076(2)
Cu3	24*k*	0	0.30860(4)	0.12068(4)	0.35(1)	0.0076(2)
Ge3	24*k*	0	0.30860(4)	0.12068(4)	0.18(1)	0.0076(2)

$^{a)}$ U_{eq} is defined as one third of the trace of the orthogonalized U_{ij} tensor

Table S2. Selected interatomic distances (Å) in $Ba_8Cu_{14}Ge_6P_{26}$.

Atom pairs		Distances (Å)	Atom pairs		Distances (Å)
		$Ba_8Cu_{14}Ge_6P_{26}$			
Ba1 –	M2× 8	3.225(1)	M1 –	M3× 4	2.324(1)
	M3× 12	3.334(1)	M2 –	M2	2.265(1)
Ba1 –	M1× 4	3.558(1)		M3× 3	2.331(1)
	M2× 8	3.734(1)	M3 –	M1	2.324(1)
	M3× 8	3.393(1)		M2× 2	2.331(1)
	M3× 4	3.862(1)		M3	2.429(1)

Note: M1 indicates the 6*c* atom site, M2 indicates 16*i* atom site, M3 indicates 24*k* atom site.

Table S3. EDS results for selected $Ba_8Cu_{14}Ge_6P_{26}$ crystals normalized to 8 Ba atoms.

Spectrum	Composition	Cu+Ge
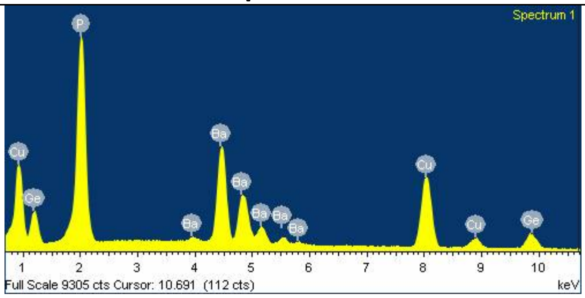	$Ba_8Cu_{14.49}Ge_{5.52}P_{22.01}$	20.01
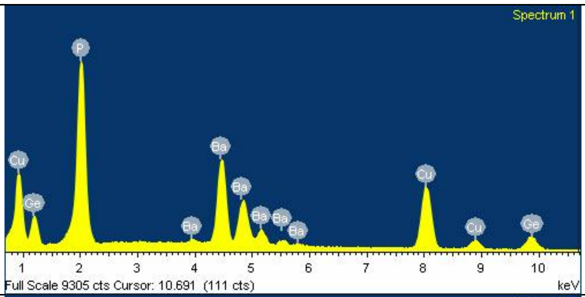	$Ba_8Cu_{14.21}Ge_{5.47}P_{21.95}$	19.68
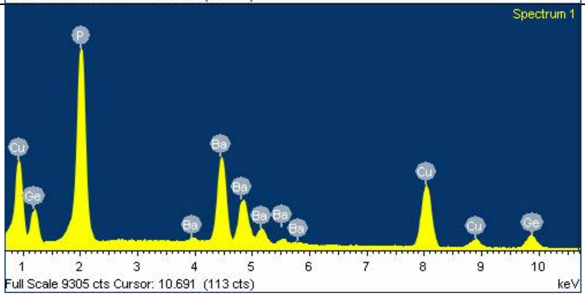	$Ba_8Cu_{14.22}Ge_{5.40}P_{23.57}$	19.62
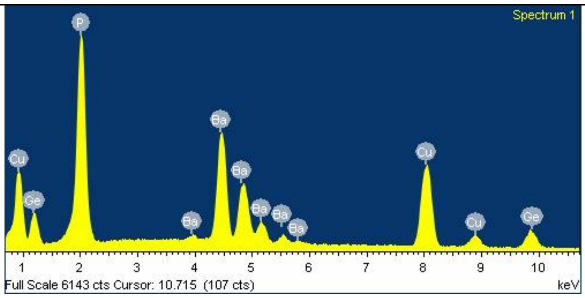	$Ba_8Cu_{14.72}Ge_{5.51}P_{19.57}$	20.23
**Averaged**	$\mathbf{Ba_8Cu_{14.4(2)}Ge_{5.5(1)}P_{22(2)}}$	**19.9(3)**

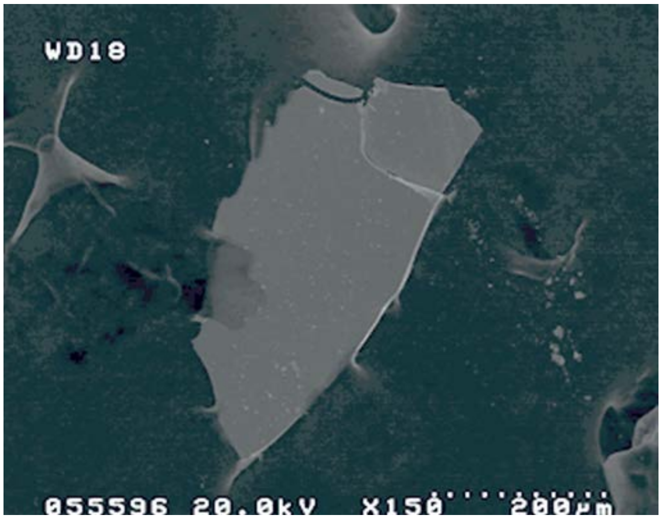

Figure S3. SEM image of $Ba_8Cu_{14}Ge_6P_{26}$ selected crystal.

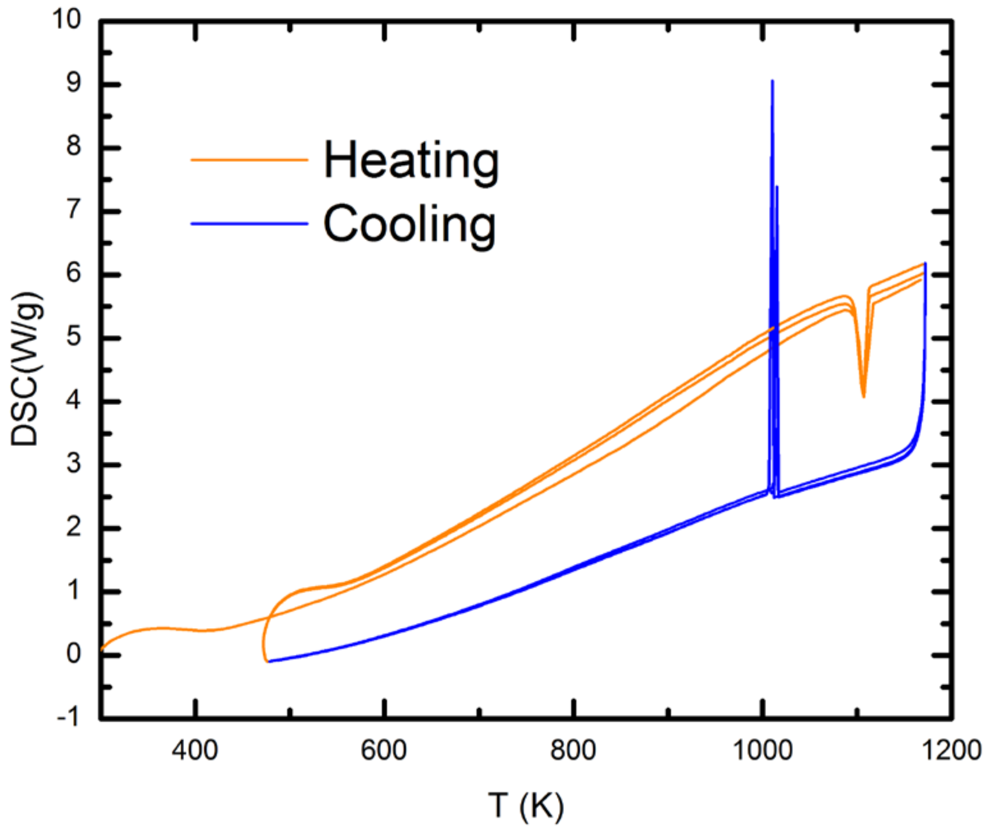

Figure S4. The DSC results of three cycles of heating and cooling of the $Ba_8Cu_{14}Ge_6P_{26}$ sample.

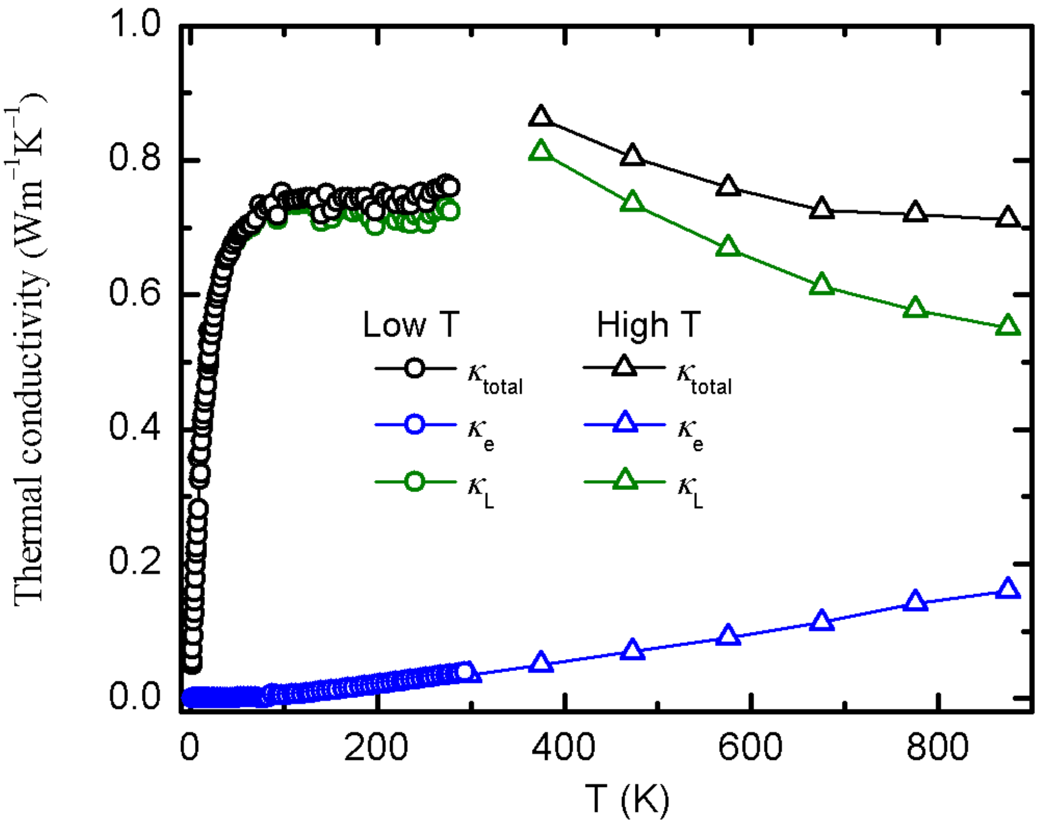

Figure S5. Thermal conductivity of the slice of $Ba_8Cu_{14}Ge_6P_{26}$ Bridgman growth crystal at low- (circles) and high-temperature (triangles) ranges. The electronic (κ_e) and lattice (κ_L) contributions to the total thermal conductivity (κ_{total}) are shown in blue and green symbols, correspondingly.

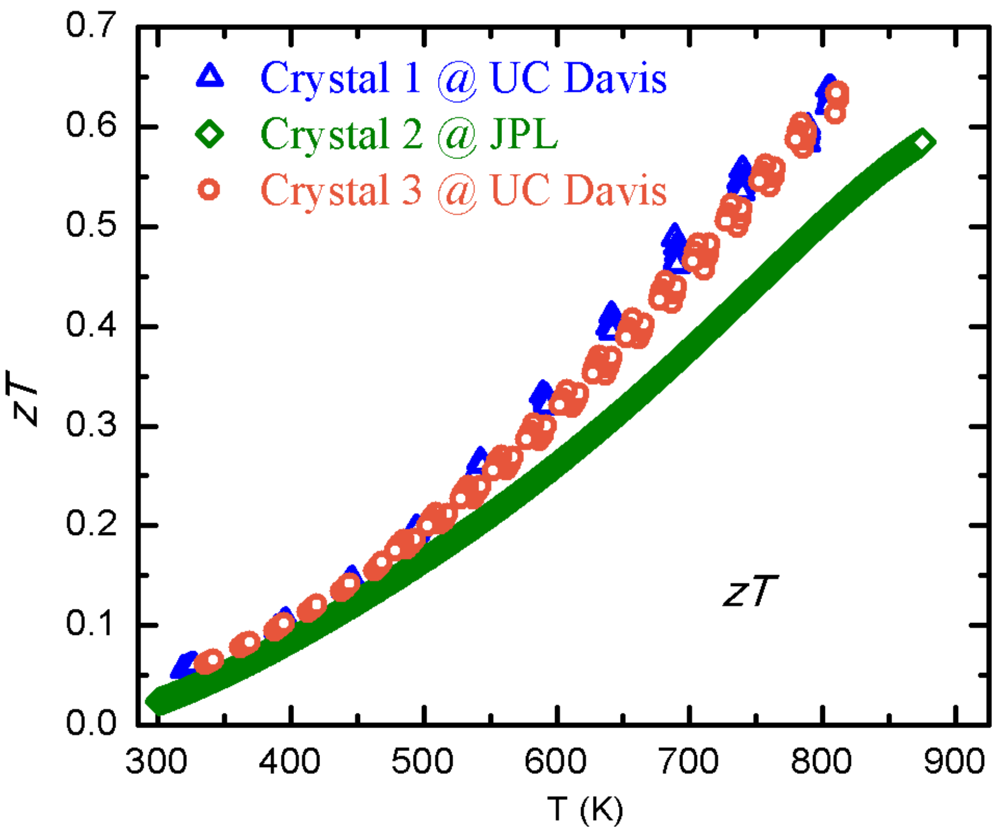

Figure S6. Comparison of thermoelectric efficiency, *zT*, of slices of three different crystals of $Ba_8Cu_{14}Ge_6P_{26}$. Crystals 1 and 3 were measured at UC Davis and crystal 2 was measured at JPL. As stated in the experimental section, the accuracy of the zT determination is ~20%. The observed difference in the *zT* might be due to different instruments and measurements methods used. See below for the detailed comparisons:

1. Borup, K.A.; de Boor, J.; Wang, H.; Drymiotis, F.; Gascoin, F.; Shi, X.; Chen, L.; Fedorov, M.I.; Müller, E.; Iversen, B.B.; Snyder, G.J. *Energy Environ. Sci.* **2015,** *8,* 423

2. Wang, H.; Bai, S.; Chen, L.; Cuenat, A.; Joshi, G.; Kleinke, H.; König, J.; Lee, H.W.; Martin, J.; Oh, M.-W.; Porter, W.D.; Ren, Z.; Salvador, J.; Sharp, J.; Taylor, P.; Thompson, A.J.; Tseng, Y.C. *J. Electron. Mater.* **2015,** *44,* 4482.

3. Alleno. E.; Bérardan, D.; Byl, C.; Candolfi, C.; Daou, R.; Decourt, R.; Guilmeau, E.; Hébert, S.; Hejtmanek, J.; Lenoir, B.; Masschelein, P.; Ohorodnichuk, V.; Pollet, M.; Populoh, S.; Ravot, D.; Rouleau, O.; Soulier, M. *Rev. Sci. Instrum.* **2015,** *86,* 011301.
